# Barriers and enablers in the management of tuberculosis treatment in Addis Ababa, Ethiopia: a qualitative study

**DOI:** 10.1186/1471-2458-8-11

**Published:** 2008-01-11

**Authors:** Mette Sagbakken, Jan C Frich, Gunnar Bjune

**Affiliations:** 1 Section for International Health, Institute of General Practice and Community Medicine, University of Oslo, P.O. Box 1130 Blindern, NO-0318 Oslo, Norway; 2 Research Unit for General Practice, Institute of General Practice and Community Medicine, University of Oslo, Norway; 3 Section for International Health, Institute of General Practise and Community Medicine, University of Oslo, Oslo, Norway

## Abstract

**Background:**

Non-adherence to tuberculosis (TB) treatment is an important barrier for TB control programs because incomplete treatment may result in prolonged infectiousness, drug resistance, relapse, and death. The aim of the present study is to explore enablers and barriers in the management of TB treatment during the first five months of treatment in Addis Ababa, Ethiopia.

**Methods:**

Qualitative study which included 50 in-depth interviews and two focus groups with TB patients, their relatives and health personnel.

**Results:**

We found that loss of employment or the possibility to work led to a chain of interrelated barriers for most TB patients. Daily treatment was time-consuming and physically demanding, and rigid routines at health clinics reinforced many of the emerging problems. Patients with limited access to financial or practical help from relatives or friends experienced that the total costs of attending treatment exceeded their available resources. This was a barrier to adherence already during early stages of treatment. A large group of patients still managed to continue treatment, mainly because relatives or community members provided food, encouragement and sometimes money for transport. Lack of income over time, combined with daily accumulating costs and other struggles, made patients vulnerable to interruption during later stages of treatment. Patients who were poor due to illness or slow progression, and who did not manage to restore their health and social status, were particularly vulnerable to non-adherence. Such patients lost access to essential financial and practical support over time, often because relatives and friends were financially and socially exhausted by supporting them.

**Conclusion:**

Patients' ability to manage TB treatment is a product of dynamic processes, in which social and economic costs and other burdens change and interplay over time. Interventions to facilitate adherence to TB treatment needs to address both time-specific and local factors.

## Background

Tuberculosis (TB) is a major public health challenge, with an estimated 8.8 million new cases and 1.7 million deaths in 2003 [1]. 27% of these cases and 31% of the deaths occur in Africa, where case rates continue to rise. The situation in Africa has been attributed to the HIV epidemic and poorly organized TB control programs [1,2]. Treatment of TB requires access to appropriate health care, but patients may find it difficult to adhere to the intended treatment even if such services are available. Studies from both low- and high-income countries show that between 20% and 50% of patients with TB do not complete treatment regimes [3,4]. Incomplete treatment may result in prolonged infectiousness, drug resistance, relapse, and death [5]. Improvement in treatment outcomes requires a better understanding of the barriers and enablers patients experience during TB treatment. Quantitative and qualitative studies from countries in Asia and Africa point to barriers related to availability and accessibility [6-8], and direct and indirect costs related to treatment [9-12]. Inconvenient routines in health care systems [13-15] and interaction with health personnel may also act as barriers [13,16-18].

In Ethiopia, where there is an increasing incidence of new infectious TB cases [19], quantitative studies from rural areas found that between 6.7% and 20% of patients interrupted treatment and that long distances to the health facilities, poor awareness about the disease and treatment length, side effects and lack of family support are important factors [20-23]. An early case control study from Addis Ababa found that "social problems" and "feeling of improvement" were major causes of treatment interruption [24]. These studies found that most patients interrupted treatment in the third or fourth month.

Few studies have used qualitative methods to explore patients' experiences during different phases of treatment, and to the authors' knowledge the studies on diagnostic delay and treatment interruption in Ethiopia have not been explored further in qualitative research.

WHO has emphasized that an important research area is identifying time points in the treatment that are of importance for different types of adherence strategy [25]. The aim of the present study is to explore enablers and barriers in the management of TB treatment during the first five months of treatment in Addis Ababa, Ethiopia.

## Methods

We used qualitative methods to explore enablers and barriers in the management of TB treatment. We found that in-depth interviews conducted during different stages of treatment were the most appropriate method for exploring how context-specific enablers and barriers interact over time. We used focus group discussions for further exploration and validation of information from the in-depth interviews. The study comprised a follow-up study with three individual in-depth interviews of 10 newly-diagnosed TB patients, in addition to one single individual interview with 11 TB patients who had interrupted treatment. We also interviewed two TB patients who were on re-treatment, four relatives, five health care staff, and conducted focus group discussions involving 11 TB patients currently undergoing treatment.

### Study setting

Data was collected over a six-month period in Addis Ababa, the capital of Ethiopia, in 2001–2002. At this time, Addis Ababa had about 95% coverage of directly observed treatment, short course (DOTS) in existing governmental health facilities [26]. TB treatment involved daily attendance for two months (the intensive phase), followed by a period of six months during which medicines were collected once or twice a month (the continuation phase). At the time of the study, HIV counselling and testing was not routinely carried out for TB patients. Anecdotal reports and clinical observations suggested a high rate of co-infections, and according to the Ethiopian Ministry of Health AIDS patients occupied about 42% of existing hospital beds [19,27]. The number of TB patients not completing their full course of treatment was difficult to estimate, as only a proportion of smear-positive patients were evaluated for their outcome [26].

### Study sites

Participants were recruited from three sites: (i) Woreda 23 Health Centre, a centre for TB diagnosis and treatment located about two kilometres from the ALERT (All African Leprosy and Rehabilitation Training Centre) hospital; (ii) St Peter's TB Specialized Hospital Outpatient Service, which offered diagnostic and treatment services and was located in the Kolfe area; (iii) and Kebele 16 Health Post, which provided TB treatment but was without diagnostic facilities (patients were diagnosed at Woreda 23 Health Centre). This health post was located in a slum area close to the ALERT hospital and training centre. (A Kebele is part of a Woreda and is the smallest administrative unit in Ethiopia.) These research sites where chosen based on advice from local researchers at Armauer Hansen's Research Institute (AHRI) and from WHO's TB/Leprosy advisor at the Ministry of Health in Ethiopia. The sites were considered to be "typical" DOT (directly observed treatment) clinics, but they were also chosen because they represented diversity being located in different areas of town. The three sites had similar routines related to daily TB treatment (DOT).

### Participants and data collection

The study included both TB patients who were attending TB treatment and TB patients who had interrupted TB treatment. Newly diagnosed TB patients ("new cases") were approached by means of an invitation letter distributed by health personnel at Woreda 23 Health Clinic. A purposeful sample of 10 patients; five men and five women aged 18 to 67 years, participated in the prospective part of the study. Two patients declined to participate because they were too ill. Participants' characteristics are displayed in Table 1. There were difficulties with tracing patients who had interrupted treatment, due to lack of, or incorrect, addresses. St Peter's TB Specialized Hospital Outpatient Service and Kebele 16 Health Post were therefore included for the purpose of recruiting patients who had interrupted treatment. We traced 11 patients that had been on treatment for at least two weeks, and had interrupted treatment for more than six consecutive weeks at the time of contact. All eleven patients agreed to participate in the study. The group consisted of six men and five women, aged 20 to 60 years. Participants' characteristics are displayed in Table 1. The National Tuberculosis and Leprosy Control Program (NTLCP) restricts its definition of defaulters to patients who have attended treatment for at least four weeks and have been absent for at least eight consecutive weeks or for a cumulative period of 12 weeks. All of our participating patients do thus not meet the formal definition of a defaulter. Focus group participants were recruited by the first author and the research assistant from all three clinics.

**Table 1:** Characteristics of 10 patients undergoing treatment and 11 patients who had interrupted treatment who were interviewed individually

**Characteristic**	**Undergoing (n = 10)**	**Interrupted (n = 11)**
	No.	No.
*Gender*		
Male	5	5
Female	5	6
*Age*		
< 25	3	2
26–35	6	6
> 36	1	3
*Education*		
Partly illiterate/illiterate	1	4
1–6 years	3	3
7–10 years	1	2
11–13 years	5	2
*Occupation*		
Daily labourer*	2	3
Civil servant	2	1
Private sector	3	4
Other	3	3

* Person who is not employed on a permanent basis, but who may meet at a regular point every day to compete with others to be hired for the day.

We developed an interview guide that covered general beliefs about TB and TB treatment, as well as time-specific factors that could affect the management of treatment. The guide was inspired by Becker's [28] review of models and strategies to find consistent predictors of adherence. Becker suggests that certain health beliefs, together with the psychological and other perceived costs of the recommended action, the interaction between patients and health service deliverers, and other types of social influence, are important dimensions for understanding adherence.

### Ethics

Informed oral or written consent was sought in all cases. De-identification and confidentiality were ensured by using numbers and fictitious names to describe and identify patients. Ethical approval for the study was obtained from the Regional Committee for Medical Research Ethics in Norway and the National Ethical Clearance Committee in Ethiopia.

### Individual in-depth interviews

The first author (MS) conducted face-to-face, tape-recorded, qualitative, in-depth interviews. The questions and answers were translated from English to Amharic and vice versa by a local research assistant/interpreter. All interviews took place at locations chosen by the patients. The interviews lasted from two to three hours. Questions in the interview guide were open-ended, and emerging themes and hypotheses from earlier interviews were explored in subsequent interviews. 10 TB patients were interviewed three times over a period of five months. The first in-depth interview was conducted two weeks after diagnosis, the second interview two months later and the third interview five months into treatment. All patients, except one who died, participated in all three interviews. A single individual in-depth interview was conducted with each of the 11 patients who had interrupted treatment. Other groups of informants, like relatives of patients that died after treatment interruption, and patients on re-treatment, were also interviewed. In-depth interviews were additionally conducted with two nurses and one doctor from Woreda 23 Health Centre, one nurse from Kebele 16 Health Post and one nurse from St Peter's TB Specialized Hospital Outpatient Service. These individuals were recruited because they were managing TB patients on a full-time basis. Participants' characteristics are displayed in Table 2.

**Table 2:** Characteristics of 5 health personnel and 6 other participants who were interviewed individually

**Characteristic**	**Health personnel (n = 5)**	**Others (n = 6)**
	**No.**	**No.**
*Gender*		
Male	3	
Female	2	
*Education*		
Nurse	4	
Medical doctor	1	
		
*Relatives of patients*		4
*Patients on re-treatment*		2

### Focus groups

Two focus group discussions with TB patients who were undergoing TB treatment were arranged after the in-depth interviews were completed. Patients with different educational backgrounds were chosen [29], yet with adjustments to personal features and the group composition as a whole. One group consisted of five female TB patients, the other of six male TB patients. Participants' characteristics are displayed in Table 3. The focus group discussions lasted for two hours, and were moderated by the research assistant, who was trained in focus group interview principles and techniques. The first author introduced pre-selected topics related to common, discrepant or particularly interesting findings from the in-depth interviews for further validation. Notes were taken, and the discussions were tape-recorded.

**Table 3:** Characteristics of 11 patients who were undergoing treatment and who were interviewed in focus groups

**Characteristic**	**No.**
*Gender*	
Male	6
Female	5
*Age*	
< 25	4
26–35	4
> 36	3
*Education*	
Partly illiterate/illiterate	2
1–6 years	3
7–10 years	4
11–13 years	2
*Occupation*	
Daily labourer	3
Civil servant	3
Private sector	2
Other	3

### Analysis

Use of an interpreter during interviewing creates a potential source of misunderstanding. We actively sought to prevent errors by continuously discussing and negotiating the content of key words, broader concepts and units of meaning. The first author and the research assistant/interpreter thoroughly discussed and clarified the content of each tape-recorded interview. Clarifying notes were added and main themes and issues to be explored in subsequent interviews or focus groups were systematically written down (conceptual maps). A "memo" (notes on each participant, including main content of the interview) was made enabling the second and third author to read about each of the participants that were interviewed and quoted in the manuscript. The research assistant translated and transcribed verbatim each interview and the discussions in the focus groups. All transcriptions were manually coded within a defined coding frame. The coding frame was developed on the basis of Becker's theories [28], and was informed by themes and issues emerging from the material. Data concerning factors that affected patients' management of TB treatment was used for systematic text condensation, according to the principles of Giorgi's [30] phenomenological analysis, modified by Malterud [31]. The analysis followed these steps: (i) reading all the material to get an overall impression; (ii) identifying units of meaning that represent different factors that influence the management of TB treatment, and coding for these; (iii) condensing and summarizing the content of each of the coded groups; (iv) integrating the insights from the condensed meaning units into generalized descriptions that reflect apparently significant factors. The analysis was summarized and accounted for in an analysis document that all authors commented on.

## Results

We found that loss of employment or the possibility to work led to a chain of interrelated barriers for most TB patients. Daily treatment was time consuming and physically demanding, and rigid routines at health clinics reinforced many of the emerging problems. Patients with limited access to financial or practical help from relatives or friends experienced that the total costs of attending treatment exceeded their available resources. This was a barrier to adherence already during early stages of treatment. A large group of patients still managed to continue treatment, mainly because relatives or community members provided food, encouragement and sometimes money for transport. Lack of income over time, combined with daily accumulating costs and other struggles, made patients vulnerable to interruption during later stages of treatment. Patients who were poor due to illness or slow progression, and who did not manage to restore their health and social status, were particularly vulnerable to non-adherence. Such patients lost access to essential financial and practical support over time, often because relatives and friends were financially and socially exhausted by supporting them. These findings are grouped into main themes and examined in further detail below.

### Loss of income

The majority of patients in the study experienced loss of employment or the opportunity to work as one of their main problems during treatment. Patients reported losing their job when their TB diagnosis was known, or because they were too ill to continue working, or were unable to find daily work because of the time consuming treatment arrangements. Three patients were housewives, but the household finances were still affected because their husbands lost income and risked their jobs escorting them to the clinic. One of the husbands explained:

"*I tried to explain for them that I was the only one working in our family, so I couldn't go every morning ... because in that way I would lose my job and me and my wife would die of hunger. I work in the private sector and it is difficult to get permission every day." (Participant 24, prospective group.)*

Patients who worked in the private sector and "daily labourers" (a person who is not employed on a permanent basis, but who may meet at a regular point every day to compete with others to be hired for the day) were most affected. Also health personnel identified these groups as particularly vulnerable:

"*People who are daily labourers or work in the private sector they can't get any kind of [sick] leave and they face more problems. Many of them interrupt treatment because they don't want to lose their jobs. They rather live for a while, with money and their jobs, to eat and then die." (Participant 27, male nurse.)*

Being ill and without access to any health care benefits often resulted in a loss of income from one day to the next. Patients' situations were aggravated because loss of income coincided with additional expenses. More than half of all the patients had been treated at private clinics before entering conventional TB treatment, paid for from savings or money borrowed from relatives or community members. Daily attendance at a clinic caused high transportation costs, and those that could not afford transportation incurred opportunity costs due to extensive time use. In addition, most patients believed that they had to eat expensive protein food like meat, milk and eggs to get cured. A daily labourer offered an illustration of how the struggle to obtain "good food" increased his already pronounced personal crisis caused by loss of income:

"*How can I get this food [good food], it is impossible to get this. I don't have a job. [...] "I'm even considering committing suicide. [...] I have lost all hope ... totally. I am the one that support me and now I can't support myself. I can't work, and then it is hard for me to survive." (Participant 10, male patient, prospective group.)*

Loss of income combined with additional costs made TB patients' particularly vulnerable during the first period of treatment (the intensive phase). Two patients in the prospective group continuously considered interrupting treatment due to a lack of money for food and transportation to the clinic.

### Hunger

Impoverishment due to loss of income caused unpredictability and emotional stress in relation to daily access to food. Many participants described feeling chronically hungry, suffering the slow starvation of people who eat a little bit every day but whose hunger is never fully satisfied. A young male patient, who lived with his single, TB inflicted mother, was too ill to continue working as a daily labourer. He related how lack of food made him lose his motivation to follow the treatment:

"*Q. During the last weeks, have you ever considered not to go to the health centre?*"

"*Yes, I thought about that many times ... on and off. Especially when I lack food I feel like not coming. [...] My only motivation is to get food ... food ... it is food that is motivating me [...] to get food, any kinds of food ... at the right time ... and to have a person that can help you get that food." (Participant 8, male patient, prospective group.)*

Chronic hunger induced a feeling of hopelessness among patients. Irregular and insecure access to food over time made many lose their hope of recovering and avoiding death.

### Taking medicines on an empty stomach

Gastritis was the most common side effect, and was reported by nearly all intensive-phase patients. The symptoms were described as a burning or gnawing pain in the abdomen. A few patients explained that they pretended to swallow the medicines, saving them until they could take them with food or milk. A female focus group participant explained:

"*I knew that I would feel the pain if I swallowed the tablets immediately [at the clinic]. Therefore I pretended swallowing the tablets. I took the tablets, hiding them. Then I swallowed the tablets later ... with milk ... at home." (Female focus group participant, focus group one.)*

Patients attributed side effects, such as gastritis, nausea, and vomiting, to taking strong medicines on an empty stomach. These side effects had a large psychological impact: Since most patients considered access to food, and particularly food with a high content of protein, as extremely important to healing, symptoms as gastritis served as a continuous reminder of their poverty and what they considered to be poor healing conditions.

### Physical demands

Most patients complained that the first two months of treatment were physically exhausting. This was particularly the case for the many patients who were at advanced stages of the disease at the time they started treatment. Patients attending treatment at Kebele 16 Health Post had shorter walking distances than those attending the other clinics. This was a tremendous advantage for the many who could not afford transportation. Most patients from the other two clinics reported using one to two hours each way. A patient with advanced symptoms, and who was also suffering from various side effects, described her daily walk to the clinic:

"*Q. Do you always walk or do you take any kind of transport?*"

*P. "I walk, I don't have any money for transport*."

"*Q. Is it hard?*"

P. "Yes, it is a bit far. I use almost two hours and I vomit on the way." (Participant 5, female patient, prospective group.)

Once at the clinic, patients often waited an hour or more to get their medication. This delay was mainly caused by intensive-phase patients being told to attend at the same time in the morning. This time-consuming system was described as humiliating by some of the patients.

### Rigid routines and health staff attitudes

The three clinics in the study practised DOT without taking into consideration that many patients were not physically able to come to the clinic. Many patients told how they had begged to be hospitalized, but due to a lack of hospital beds this option was out of the question for many. One patient died on the 59th day of treatment, having been refused medical attendance before the 60th day, in accordance with clinic routines. Her husband described how they had struggled to make health personnel understand that she was far too ill to walk to the clinic:

"*We went to the clinic to ask if the nurse could give her the medicines, at least once in seven days, telling him that our house was far away [...] and explaining them that she was too weak to go to the clinic every day* ... *she couldn't walk at that time. The nurse didn't understand, rather he threatened her saying he would demand her to come and collect the medicines no matter how ill she was." (Participant 24, prospective group.)*

In a few cases, relatives were allowed to collect the medicines on behalf of very ill patients. This, however, put the relatives' jobs in jeopardy, as they had to queue at the clinic every day. In most cases, the patients were escorted to the clinic by relatives. This incurred transportation and opportunity costs for two persons, often within the same household. The clinics allowed no exceptions for family- or cultural events, and were highly inflexible as regards patients trying to combine daily treatment with work-related activities. Only one patient in the prospective group managed to hold on to his job. He explained the repeated difficulties he faced trying to balance the demands of work with the treatment arrangements:

"*Once I came late and I met the bad nurse ... and he was angry about it. He told me to sit and wait for him until he got back from town, just to give me my medicines. After he warned me not to come late anymore and then he gave me the medicines. He said, 'next time you will be given the injections even later and you will be very late for your work'." (Participant 6, male patient, prospective group.)*

Some nurses were more flexible than others, but there were examples from all three clinics of patients who were threatened, humiliated or treated angrily by staff for not adhering to the implicit rules of the system. At one of the clinics, two patients reported that they had been denied access after a period of interruption.

### Dynamics of social support

The support of family and community members was extremely important during the intensive phase of treatment, because this to a large extent compensated for loss of income. Even though TB caused fear and stigmatization, people shared resources in a time of crisis, and reciprocal arrangements provided most TB patients with some food. Many received physical support in walking to the clinic, and some received money for transportation. A male patient who lost his job as a car washer explained how the contributions of relatives and community members helped him:

"*Even if I can't get good food I try to get as much food as possible. People who visit me bring me food ... neighbours and relatives." (Participant 9, male patient, prospective group.)*

Many patients experienced changes in the level of support as the treatment program progressed. A male patient, who lost his job as a temporary teacher, moved to live with relatives in a rural area after completing the intensive phase. His friends and grandmother no longer had the means to help him:

"*She [grandmother] couldn't afford to help me anymore. She couldn't help me because of her shortage. They are all fed up [...] I have moved, walked from place to place ... and there is no food. In the rural area, even if my father is poor, I at least managed to get milk from the neighbourhood." (Participant 1, male patient, prospective group.)*

Many patients faced difficult situations after completing the intensive phase of treatment, as the levels of support dropped. Both patients and health personnel reported that the causes of the changes in support levels were closely related to practical and symbolic changes in treatment at this stage.

### Crisis precipitated due to completion of the intensive phase

Based on a synthesis of the various sources of data we found completion of the first phase of treatment to precipitate changes, often critically affecting existing support mechanisms: First, patients were expected to stop going for treatment on a daily basis, and were not expected to need money for transport or help with walking to the clinics. These changes reduced the attention focused on their difficulties, and their needs therefore became less visible. Second, it was expected that the health status of patients who had finished the streptomycin injections, which were considered the "main" medicine, should improve significantly. Third, physical improvement was associated with the expectation that patients would start to work again. Fourth, the foods provided from the community were expected to make a physical difference in patients. There is a strong association between TB and HIV/AIDS, and patients with a "curable disease" are expected to gain weight and strength within a certain period of time. Patients with prolonged disease (intermittent symptoms or no weight gain) may be treated as having an incurable disease (AIDS or a "chronic" type of TB), a condition that carries additional stigma.

Six out of 10 patients in the prospective group, all still financially dependent on others, experienced increasing problems in mobilizing enough help in the period between the second and fifth months. Some gradually received less food, others were told to leave the house because they were no longer able to contribute with the rent. The experience of one patient, who still displayed symptoms as skinniness and weakness during the continuation phase, is illuminating. He reported how his relationship with his brother and sister-in-law became increasingly tense because he could no longer pay his share of the food and rent. He was told to leave in his fifth month of treatment:

"*People will get bored of you. Earlier they supported me when they had money, or with some food, but they don't do that much more. I can't ask for help all the time. (Crying) It is at home ... they have asked me to leave their house now." (Participant 10, male patient, prospective group.)*

Relatives and community members may start to interpret the slow recuperation process negatively, as indicating eventual death, or they may assume that the patient will not mange to re-establish his or her income. The basis of reciprocity is taken away both when a patient become totally dependent either permanently or for a prolonged period of time, and when people think that the patient is dying. When health personnel were asked about their experiences related to treatment interruption, they all mentioned patients leaving to live with relatives in rural areas as one of the main causes. This was seen as a consequence of patient-relative relationships being financially or emotionally exhausted in the later stages of treatment.

### Interplay of factors

Attending TB treatment brought with it various struggles to meet the physical, psychological and financial costs of treatment. Interviews with patients who had interrupted treatment also demonstrated the interplay of such factors. Five patients interrupted during the intensive phase of treatment, two of them with only a few days left. Six patients interrupted between the third and sixth months of the continuation phase. Only one patient pointed to a single factor as the cause of treatment interruption. This patient was denied a sufficient ration of medicine to enable her to travel to the place where her son just had been murdered. The causes of interruption were different, but interrelated. They often operated over time and were most often related to financial constraints. Three examples that illustrate identifiable patterns in treatment interruption are presented below.

#### Case one

A single mother, a working immigrant from a rural area, used to beg outside one particular church before and after morning mass. She had a small but regular income from regular church visitors. Due to DOT, which takes up the morning hours, she couldn't do that anymore, and her only source of income was dramatically reduced. As a result, she was unable to pay for the room she rented, and she and her baby ended up on the street. She turned to the health personnel, telling them that she was starving and that she needed to take the medicines with her to her mother in the countryside. Her request was denied, and she decided to leave without the medicines after two weeks of treatment:

"*I didn't intend to interrupt the medicines at that time, but I was too poor to keep on taking the medication. I was told to eat eggs, meat and to drink milk, but the truth was *... *I didn't even have injera [Ethiopian pancake]. I went to the countryside because I was forced to go there." (Participant 19, female patient, retrospective group.)*

This case illustrates how poor patients without rights to paid sick leave or access to financial support from family and friends experience acute and unbearable financial crises during early stages of treatment.

#### Case two

A male TB patient interrupted treatment shortly before completing the intensive phase. During treatment, he made intensive efforts to find daily work, but his physical condition and the time-consuming system made this difficult. He often had no money for transport, and he felt weak and tired from having to walk four kilometres twice a day. Sometimes he was late for treatment, which made the health personnel angry. After about a month and a half, he experienced that the friends who had helped him the most became more reluctant to support him with money. At the same time his relatives, all living in a rural area, invited him to a funeral. He asked the health personnel to be allowed to attend, but this request was denied. He decided to leave, interrupting treatment. When asked why he interrupted, he gave several reasons:

"*P. I didn't finish the treatment because I had to work to get food and I was told to eat anything possible for the medication and for the disease ... otherwise the medicines can't work ... and to get food I had to work. The other thing was that I didn't have money for transport to come to the clinic and I couldn't walk always because I would be too late by the time I arrived at the clinic. [...] I felt weak by walking to the clinic everyday, and the insult of health personnel discouraged me*.

Q. Would you say this whole situation made you go to your relatives?

P. I went there because I was obliged to go there. (Pause) But I was not motivated to come on foot to the hospital anymore." (Participant 13, male patient, retrospective group.)

This case illustrates the situation where a patient ends up in a vicious circle, where the demands of treatment become more and more difficult to handle. A funeral or a sick relative then represents a breaking point; a legitimate way out of daily and accumulating struggles.

#### Case three

A young girl interrupted treatment after four and a half months. At the time she started the medication, she was too ill to walk to the clinic, and her mother collected the medicines every day on her behalf. The time the mother spent on daily attendance and nursing her daughter made it impossible for her to work, and both mother and daughter became totally dependent on food provided by their neighbours. After two months of daily treatment, the community members expected them to manage on their own again. However, re-establishing herself as a beggar and laundry washer proved difficult for the mother, and as she was the only breadwinner, she became unable to feed all her children. Desperation and hunger made the sick girl break with the established social norms of the community by begging in her own neighbourhood. This led to unbearable fights with her mother:

"*I started with the treatment, but after a while I felt so hungry and the shortage of food at home *... *and because I felt like mentally disturbed taking the medication *... *made it impossible for me to continue. On top of that my mother treated me so badly at home. To overcome the shortage of food at home I started to beg food and money from the neighbourhood. This begging thing made my mother really mad. She said I was humiliating her. We had some really big fights and after that I escaped from home." (Participant 21, female patient, retrospective group.)*

This case illustrates how prolonged illness may affect relations with relatives and community members. Weeks or months of involuntary dependency force patients into humiliating situations, while relatives and friends may suffer from social or financial exhaustion.

## Discussion

### What does this study add to previous knowledge?

This study suggests that patients' management of treatment is a product of dynamic processes and that patient's behaviours respond to the complex patterns of socio-economic and psychological factors during the course of treatment.

The effect of time per se on adherence behaviour is described by Christensen-Szalanski and Northcraft [32]. They point to the importance of understanding patients' temporal perception of costs and benefits, and how temporal perceptions may be influenced by delayed benefits and accrued costs. We found that most patients experience a chain of interrelated barriers during the intensive phase of treatment. This chain is often activated by loss of income and followed by lack of money for transport; extensive time use; daily physical demands; food insecurity; side effects and hunger. Rigid routines related to daily attendance exacerbate many of these problems. TB patients loosing their jobs and the opportunity to work has been reported both in other parts of Ethiopia and in other countries [6,12-14,33,34]. A study from Addis Ababa [35] reported walking distances up to two hours and found that the distances from patients' homes to the health centres contributed to diagnostic delay. The author underlines that the same factor may seriously affect TB patients' ability to attend daily treatment.

We found that barriers in the intensive phase are managed by most patients, albeit in different degrees, partly determined by the extent of financial and practical support from family and neighbours. The protective effects of family support have been found in other studies [6,10,33,36]. One study indicates that initially strong family support became weaker during treatment [14]. Our study suggests that patients who have fewer human or material resources available in their environments, such as the very poor, single mothers, and working immigrants from rural areas, do not benefit from such protective factors. Some of these patients may be forced to change their strategy and, as related in this study, forced to move in with relatives in other areas due to acute financial crisis that arise even during the early stages of treatment.

We found that a decision to interrupt TB treatment may be taken in response to social obligations, such as attending a funeral. A study from Addis Ababa [24] found illness of relatives and attendance at funerals to be two of the most frequently cited reasons for treatment interruption. Even if such decisions are triggered by strong social norms, they may be shaped by past struggles and accrued costs, which may seem unbearable at the moment the decision is taken. Hungry patients who lack the strength or motivation to continue treatment have also been found elsewhere [37]. Previous studies from Ethiopia have found that most patients interrupt in their third or fourth month of treatment [22-24]. One study [23] found lack of family support to be one of the major causes of interruption, and this is supported by the findings in this study: Many patients ended up in a vicious circle, where poverty and possible co-infections (HIV) led to slow recovery, and where slow recovery contributed to income poverty and further dependency. These barriers manifested themselves at a time when psychological and other costs had accumulated, and enablers like family and community support were decreasing. This study shows that illness-induced poverty over time threatens the sustainability of household finances, as well as the social relations that serve as the basis of the household's continued existence as a social unit.

Our findings suggest that it is important that the health care system is aware of the dynamics of social support mechanisms. In Ethiopia there seems to be a change during treatment from a "generalised" to a more "balanced" type of reciprocity. This movement seems to be consistent with symbolic and practical changes related to completion of the first stage of treatment (the intensive phase), but may also be a product of increasing HIV/TB co-infection rates. In people's experiences, patients with prolonged diseases or intermittent symptoms often die, and social support may therefore now be withdrawn earlier than before.

### Validity and transferability

The trustworthiness of the study and validity of our findings is strengthened by the extensive triangulation within the study. We interviewed different groups of patients, health professionals and patients' relatives. The same themes were investigated from both a prospective and a retrospective angle, by means of different methods. Our findings are characterized by similarities within and between different groups of respondents and we believe this strengthen the internal validity of our study.

The sample size is limited and restricted to one geographical area, and instead of proposing that the findings are transferable to other contexts, we suggest using the concept of "extrapolation" [38] to describe the usefulness of our study. Patterns of findings presented in a broad contextual frame enable us to be aware of potential similarities in similar contexts, in particular how variables may interrelate at time-specific points in treatment and how certain identifiable patterns may create barriers in relation to the management of treatment.

TB control managers and others may argue that by not strictly following NTLCP's operational definition of a defaulter the findings will be less applicable. We believe the implications are few since the strict definition is mainly used to separate between defaulters who re-enters treatment being smear positive again, and patients that may recommence treatment to finish the initial regimen. An important implication though may be increasing awareness of patients that may "default" due to poverty related barriers early in treatment (< 4 weeks), but that are not registered as defaulters. The criteria used for defining defaulters may result in relatively low overall rates of default, also pointed to in another study from Ethiopia [23].

### Implications for practice

To our knowledge there are few major changes in how the DOT services are organized in Addis Ababa. One change that has been made is the establishment of a formal collaboration between services related to TB and services related to HIV/AIDS [39]. However, a recent study examining the acceptability of HIV testing among TB patients, conclude that the acceptability is low, and that it poses a challenge to the scale-up of TB/HIV collaborative efforts [40]. The findings in the present study may help to predict treatment interruption during the early stages of treatment, based on patients' socioeconomic and environmental conditions. Some of the barriers patients experience during the intensive phase could easily be solved with more flexible approaches, such as more flexible hours for attendance and greater flexibility related to participation in important cultural and family-related events. A primary nurse-patient system, where patients are followed up by the same nurse, would help health personnel to identify each patient's resources and constraints. More individual approaches will make it easier to establish relations based on trust, and will facilitate flexible adjustments by means of follow-up talks throughout treatment.

We believe that TB programs need to address nutrition. As recognized by many patients, without food medical treatment is in vain. Providing one glass of milk or a small meal at the clinic could have a positive influence on both case detection rates and treatment completion. By ensuring access to some nutritious food, patients may gain weight and recover faster, probably the best way to fight stigma and to strengthen social support mechanisms during later stages of treatment. Initiating a dialogue with labour organizations to help prevent TB patients losing their job is another important step.

Still, many patients will experience difficulties related to daily attendance. Further decentralization of TB-related health care, use of mobile clinics, the provision of transport money and permitting some of the poorest or most ill patients to take tablets home for self-treatment may be means to increase adherence. A recent review of randomized controlled trials, comparing DOT with self-administration of therapy, provides no evidence that the routine use of DOT in low- and middle income countries improves cure or treatment completion in people with tuberculosis [5]. Several authors have advocated a shift in perspective, where patients' socioeconomic environments, their well-being's and their dignity are included in future strategies [2,41,42]. Strategies based on self-treatment can be strengthened by support and supervision by an identified relative or neighbour, or through other social structures. Several TB control programmes that leave the choice of DOT supervisor to the patient have been shown to be successful [43-45].

In order to enable patients to get well, one need to make an assessment of what formal and informal sources of support exist, what structures are missing, and how different contributors can fill the gap [2]. In Ethiopia, the involvement of social support structures beyond relatives seems to be important. One useful structure could be the *edir*, a community based body with social responsibility (mainly for arranging funerals) for those living within a defined community (Kebele). The *edir *could become involved through the appointment of persons to be in charge of supporting TB patients who are unable to attend treatment on a daily basis. Social support structures could also be established based on the model of the successful "TB clubs", which are small groups of patients who live near each other, implemented in some rural parts of Ethiopia. These clubs, which collaborate with health workers and key persons in the community, have become an integral part of the tuberculosis program by referring suspected cases, promote treatment adherence and trace patients who interrupt [33,46]. Existing community mechanisms can be strengthened by involving the communities and creating a climate of awareness and shared responsibility for solving major health issues. By involving the communities, more flexible arrangements can be facilitated, the treatment burden can be reduced, and a better balance can be ensured between control measures and enabling measures.

## Conclusion

Patients' ability to manage TB treatment is a product of dynamic processes, in which various social and economic costs and other burdens change and interplay over time. Interventions to facilitate adherence to TB treatment need to address both time-specific and local factors.

## Competing interests

The author(s) declare that they have no competing interests.

## Authors' contributions

MS initiated the research, wrote the research proposal, conducted the research, analyzed the data and wrote this paper. JCF contributed to the conception and design of the study and the analysis and interpretation of the data. He also contributed to the writing of this paper by critically revising it. GB contributed to the conception and design of the study. He also contributed to the writing of this paper by critically revising it. All authors read and approved the final manuscript.

## Pre-publication history

The pre-publication history for this paper can be accessed here:


